# Supplementary material for: Longitudinal assessment of COVID-19 vaccine immunogenicity in people with HIV stratified by CD4+ T-cell count in the Netherlands: A two-year follow-up study
Source: PLoS One. 2025 May 19;20(5):e0323792. doi: 10.1371/journal.pone.0323792 (PMC12087993; doi:10.1371/journal.pone.0323792)
Supplement: S1 Table — (DOCX) [file pone.0323792.s001.docx]

**S1 Table. Overview of national COVID-19 vaccination recommendations for PWH.**

| **Country** | **COVID-19 vaccination recommendations for immunocompromised individuals**^*^ | **Eligibility criteria for vaccination in people with HIV** | **Recommendation based on a CD4+ T-cell count < 200 cells/µL** | **Source** | **Last update** |
| --- | --- | --- | --- | --- | --- |
| USA | Two doses of 2024-2025 vaccine spaced 6 months apart, with additional doses possible under shared clinical decision-making. | Advanced HIV infection (people with HIV and a CD4+ T-cell count < 200/mm^3^, history of an AIDS-defining illness without immune reconstitution, or clinical manifestations of symptomatic HIV) or untreated HIV infection. | Yes | Centers for Disease Control and Prevention | 31 October 2024 |
| Canada | Two vaccine doses per year. | HIV with AIDS-defining illness or TB diagnosis in last 12 months before starting vaccine series, or severe immune compromise with a CD4+ T-cell count < 200 cells/µL or CD4% < 15%, or without HIV viral suppression. | Yes | Public Health Agency of Canada | 5 February 2025 |
| United Kingdom | Additional doses, usually offered on the NHS in spring and early winter. | HIV infection. | No | National Health Service England | 2 September 2024 |
| The Netherlands | Annual vaccination in autumn, with additional doses possible based on the treating physician’s recommendation (individualised approach). | HIV infection. | No | Rijksinstituut voor Volksgezondheid en Milieu | 2 January 2025 |
| Germany | Annual vaccination in autumn. | HIV infection. | No | Robert Koch Institute | 23 January 2025 |
| France | Two vaccine doses per year, in autumn and spring. | HIV infection not specified. | No | Haute Autorité de Santé | 27 February 2025 |
| Spain | Annual vaccination in autumn-winter. | HIV infection.  People living together with a person with HIV who has a CD4+ T-cell count < 200 cells/µL. | No | Spanish Ministry of Health, Comisión de Salud Pública | 18 July 2024 |
| Sweden | No general recommendation. Treating physicians determine the need for vaccination for immunocompromised individuals. Most people with some form of immunodeficiency are recommended annual vaccination in autumn-winter. | HIV infection not specified. | No | Public Health Agency of Sweden Folkhälsomyndigheten | 6 February 2025 |
| Australia | People aged 18-74 years are recommended a booster dose every 12 months and eligible for a dose every 6 months. Those aged 75 years and older are recommended a booster dose every 6 months. | HIV with a CD4+ cell count < 200 cells/µL. | Yes | Australian Government Department of Health and Aged Care | 20 November 2024 |

^*^ After completion of a primary COVID-19 vaccination series.
