# Supplementary material for: Longitudinal assessment of COVID-19 vaccine immunogenicity in people with HIV stratified by CD4+ T-cell count in the Netherlands: A two-year follow-up study
Source: PLoS One. 2025 May 19;20(5):e0323792. doi: 10.1371/journal.pone.0323792 (PMC12087993; doi:10.1371/journal.pone.0323792)
Supplement: S2 Table — The association between participant-related and vaccine-related variables on the magnitude of spike (S1)-specific IgG antibodies overall, and how the association of these variables with S1-specific IgG antibodies changes over linear and quadratic time, was evaluated by a linear mixed-effects model. (DOCX) [file pone.0323792.s002.docx]

**S2 Table. Linear mixed-effects model of variables associated with S1-specific antibody levels over time.** The association between participant-related and vaccine-related variables on the magnitude of spike (S1)-specific antibodies overall, and how the association of these variables with S1-specific antibodies changes over linear and quadratic time, was evaluated by a linear mixed-effects model.

|  | **Estimate** | **Standard Error** | **p-value** |
| --- | --- | --- | --- |
| Time | 0.001 | 0.001 | 0.35 |
| I(time^2) | < -0.001 | < 0.001 | 0.15 |
| CD4+ T-cell count < 200 | -1.18 | 0.15 | < 0.001 |
| Male sex | -0.04 | 0.08 | 0.63 |
| Age | -0.008 | 0.002 | < 0.001 |
| Nadir CD4+ T-cell count | < -0.001 | < 0.001 | 0.21 |
| Vector-based primary vaccination | -0.39 | 0.07 | < 0.001 |
| S1-specific antibodies one month after the primary vaccination series | < 0.001 | < 0.001 | < 0.001 |
| Time since the last COVID-19 vaccination dose or SARS-CoV-2 infection | -0.01 | < 0.001 | < 0.001 |
| Time: CD4+ T-cell count < 200 | 0.002 | < 0.001 | 0.03 |
| Time: Male sex | < -0.001 | < 0.001 | 0.97 |
| Time: Age | < 0.001 | < 0.001 | 0.006 |
| Time: Nadir CD4+ T-cell count | < 0.001 | < 0.001 | 0.28 |
| Time: Vector-based primary vaccination | 0.001 | < 0.001 | 0.01 |
| Time: S1-specific antibodies one month after the primary vaccination series | < 0.001 | < 0.001 | < 0.001 |
| Time: Time since the last COVID-19 vaccination dose or SARS-CoV-2 infection | < 0.001 | < 0.001 | < 0.001 |
| I(time^2^): CD4+ T-cell count < 200 | < -0.001 | < 0.001 | 0.14 |
| I(time^2^): Male sex | < 0.001 | < 0.001 | 0.65 |
| I(time^2^): Age | < 0.001 | < 0.001 | 0.11 |
| I(time^2^): Nadir CD4+ T-cell count | < 0.001 | < 0.001 | 0.18 |
| I(time^2^): Vector-based primary vaccination | < -0.001 | < 0.001 | 0.09 |
| I(time^2^): S1-specific antibodies one month after the primary vaccination series | < 0.001 | < 0.001 | < 0.001 |
| I(time^2^): Time since the last COVID-19 vaccination dose or SARS-CoV-2 infection | < 0.001 | < 0.001 | < 0.001 |
