# Supplementary material for: Longitudinal assessment of COVID-19 vaccine immunogenicity in people with HIV stratified by CD4+ T-cell count in the Netherlands: A two-year follow-up study
Source: PLoS One. 2025 May 19;20(5):e0323792. doi: 10.1371/journal.pone.0323792 (PMC12087993; doi:10.1371/journal.pone.0323792)
Supplement: S3 Table — (DOCX) [file pone.0323792.s003.docx]

**S3 Table. Characteristics of the four participants who received treatment for a SARS-CoV-2 infection.**

|  | **Sex** | **Age** | **Most recent CD4+ T-cell count,** cells per µL | **Time since last COVID-19 vaccination dose,** months | **Last measured S1-specific antibody level,** BAU per mL | **Dominant variant at the time of the SARS-CoV-2 infection** | **Treatment** |
| --- | --- | --- | --- | --- | --- | --- | --- |
| A | Male | 61 | < 350 | 3 | 353 | Omicron | ICU admission |
| B | Male | 80 | ≥ 500 | 1.5 | 88 | Delta | Hospital admission, oxygen treatment, and corticosteroids |
| C | Male | 64 | < 500 | 6 | 415 | Delta | Hospital admission and oxygen treatment |
| D | Male | 75 | ≥ 500 | 3 | 1860 | Omicron | nirmatrelvir/ritonavir treatment |
