# Supplementary figures and images for: Longitudinal assessment of COVID-19 vaccine immunogenicity in people with HIV stratified by CD4+ T-cell count in the Netherlands: A two-year follow-up study
Source: PLoS One. 2025 May 19;20(5):e0323792. doi: 10.1371/journal.pone.0323792 (PMC12087993; doi:10.1371/journal.pone.0323792)

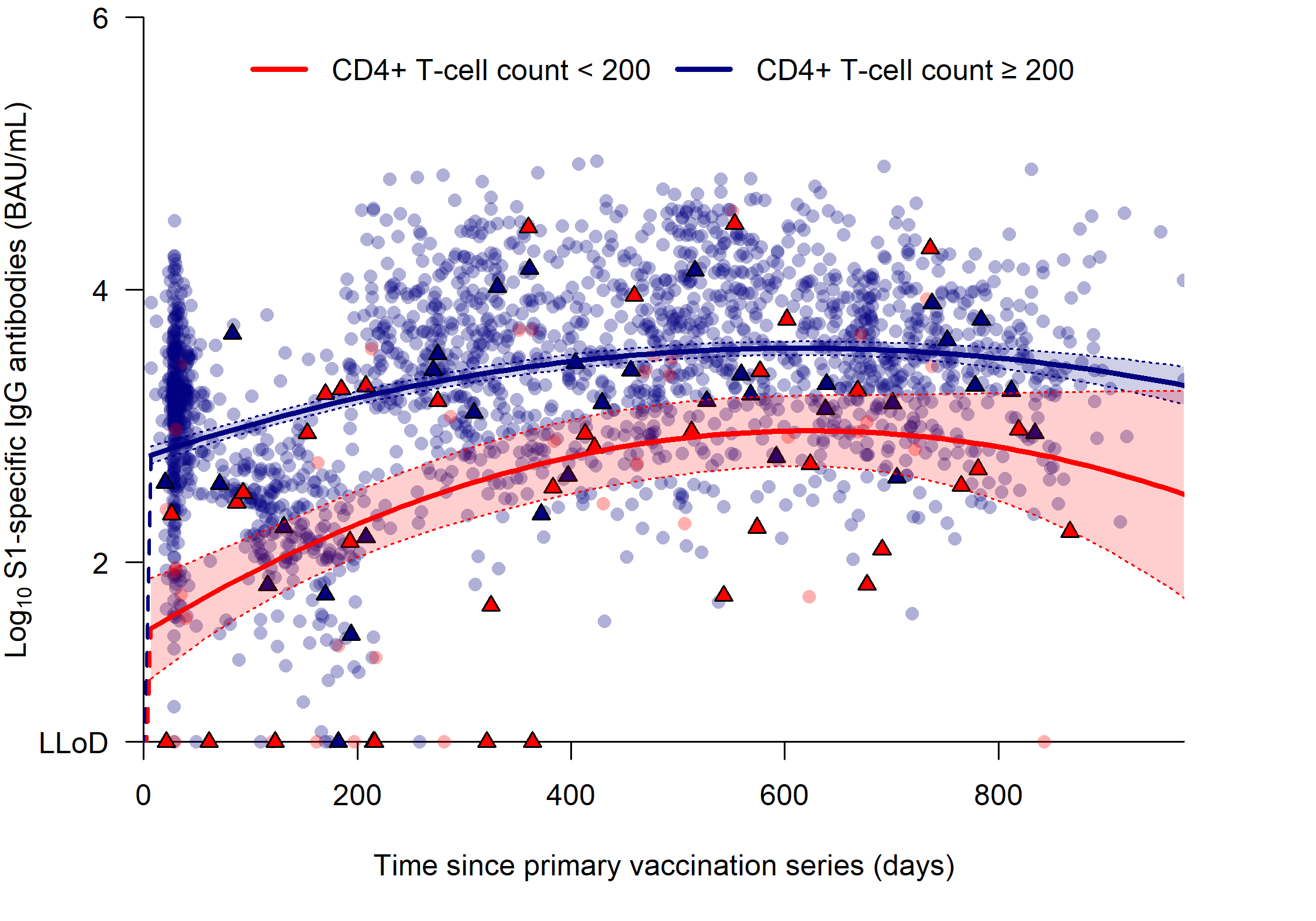

Supplement: S1 Fig — The 16 retrospectively included PWH are denoted by triangles (n = 8 with a CD4+ T-cell count < 200 cells per µL; dark red triangles, and n = 8 with a CD4+ T-cell count ≥ 200 cells per µL; dark blue triangles). Comparison between PWH with a CD4+ T-cell count < 200 cells per µL (n = 16; red) and PWH with a CD4+ T-cell count ≥ 200 cells per µL (n = 432; blue) was performed using the likelihood ratio test. The solid lines represent the mixed-effects regression of the log10 S1-specific IgG antibody levels per baseline CD4 group over time, with the dotted lines representing the 95% confidence interval. Abbreviations: BAU, binding antibody unit; LLoD, lower limit of detection; S, spike. (TIF) [file pone.0323792.s004.tif]

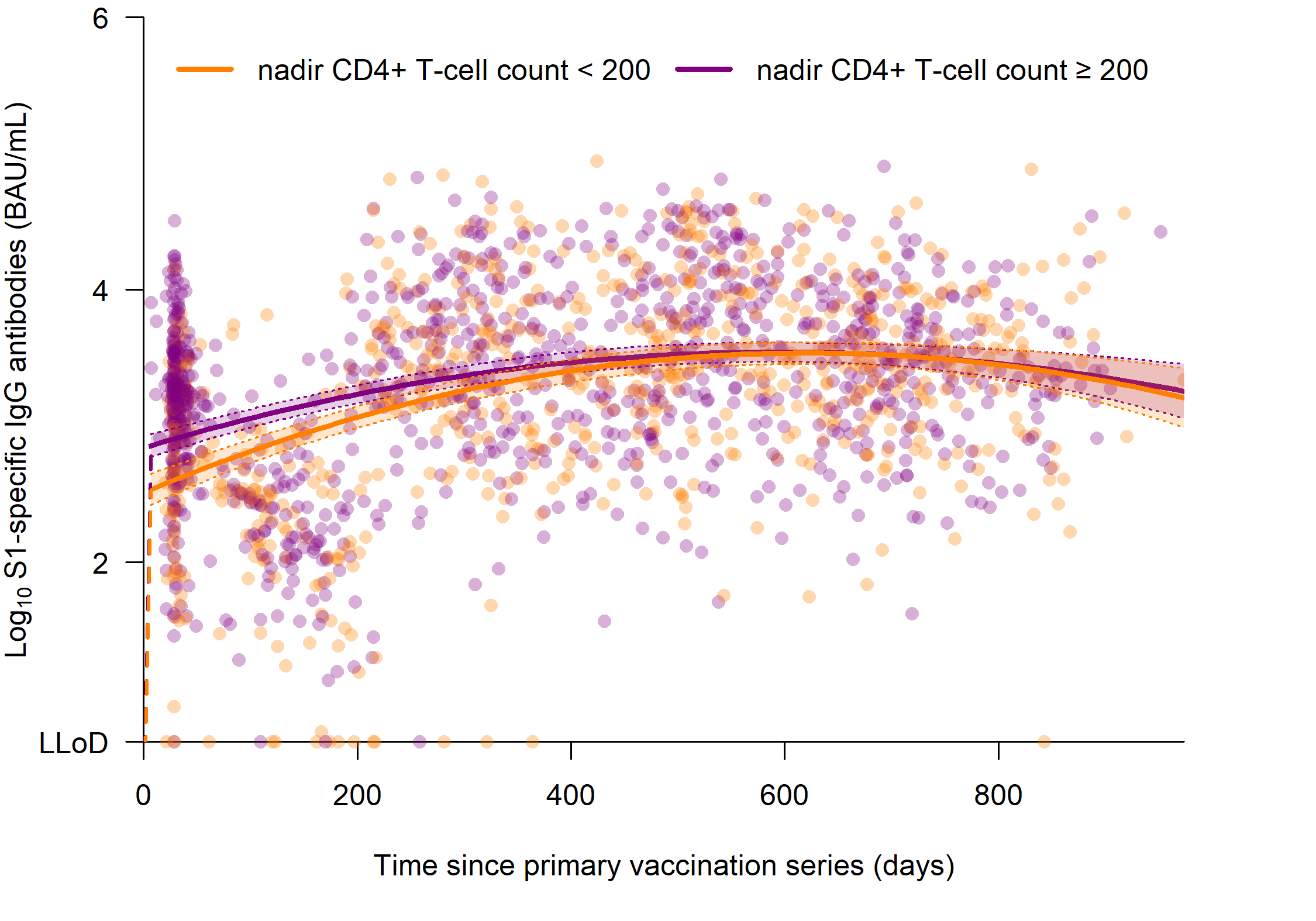

Supplement: S2 Fig — Comparison between PWH with a nadir CD4+ T-cell count < 200 cells per µL (n = 163; orange) and PWH with a nadir CD4+ T-cell count ≥ 200 cells per µL (n = 210; purple) was performed using the likelihood ratio test. The solid lines represent the mixed-effects regression of the log10 S1-specific IgG antibody levels per nadir CD4 group over time, with the dotted lines representing the 95% confidence interval. Missing data: nadir CD4 + T-cell count was unknown for 75 PWH. Abbreviations: BAU, binding antibody unit; LLoD, lower limit of detection; S, spike. (TIF) [file pone.0323792.s005.tif]
