# Supplementary material for: Longitudinal assessment of COVID-19 vaccine immunogenicity in people with HIV stratified by CD4+ T-cell count in the Netherlands: A two-year follow-up study
Source: PLoS One. 2025 May 19;20(5):e0323792. doi: 10.1371/journal.pone.0323792 (PMC12087993; doi:10.1371/journal.pone.0323792)
Supplement: Attachment — Submitted filename: PONE-D-24-47904-Nov-16.pdf [file pone.0323792.s006.pdf]

PLOS ONE

Longitudinal assessment of COVID-19 vaccine immunogenicity in people with HIV stratified by CD4+ T-cell count in the Netherlands: a two-year follow-up study

--Manuscript Draft--

**Manuscript Number:**	PONE-D-24-47904
**Article Type:**	Research Article
**Full Title:**	Longitudinal assessment of COVID-19 vaccine immunogenicity in people with HIV stratified by CD4+ T-cell count in the Netherlands: a two-year follow-up study
**Short Title:**	Two-year follow-up of COVID-19 vaccine immunogenicity in people with HIV stratified by CD4+ T-cell count
**Corresponding Author:**	Anna Helena Roukens, M.D., Ph.D.<br>Leiden University Medical Center: Leids Universitair Medisch Centrum<br>Leiden, NETHERLANDS, KINGDOM OF THE
**Keywords:**	COVID-19 vaccine response, people with HIV, CD4-cell count, long term vaccine response
**Abstract:**	Background: Guidelines for COVID-19 additional vaccination strategies for PWH are inconsistent worldwide, with some recommending an annual vaccination for all PWH, while others restrict this to PWH with a CD4+ T-cell count <200 cells per µL.<br>Methods: We conducted a prospective cohort study in 448 adult PWH. The primary outcome was the SARS-CoV-2 spike (S1)-specific antibody level at 1, 6, 12, 18, and 24 months after completing a primary COVID-19 vaccination series. We compared the antibody kinetics over two years between PWH with a baseline CD4+ T-cell count <200 (n=16) vs. ≥200 (n=432) with a mixed-effects model. Secondary outcomes included variables associated with the kinetics of S1-specific antibody levels and the incidence of breakthrough infections.<br>Results: The median most recent CD4+ T-cell count was 140 (IQR 80–165) in the <200 group, and 688 (IQR 520–899) in the ≥200 group at the time of primary vaccination. S1-specific antibodies were lower in PWH with a CD4+ T-cell count <200 vs. ≥200 during the two-year follow-up, with predicted S1-specific antibody levels of 514 (95% CI 456–578) vs. 2758 (95% CI 1488–5110) BAU/mL at 12 months (p < 0.00001) and 839 (95% CI 732–959) vs. 3505 (95% CI 1712–7175) BAU/mL at 24 months (p = 0.0001). The overall incidence of SARS-CoV-2 infections was 55% and comparable between groups. A CD4+ T-cell count <200, higher age, and a vector-based primary vaccination regimen were negatively associated with S1-specific antibody levels over time.<br>Conclusion: Long-term humoral responses were lower in PWH with a CD4+ T-cell count <200 compared to those with a CD4+ T-cell count ≥200. National COVID-19 vaccine guidelines recommending booster vaccines for all PWH, should therefore specifically emphasize the need for booster vaccines in those with a CD4+ T-cell count <200 cells per µL.
**Order of Authors:**	Marlou J Jongkees, M.D.
	Susanne Bogers
	Rory D de Vries
	Corine H GeurtsvanKessel
	Pedro Miranda Afonso
	Kathryn S Hensley
	Bart JA Rijnders
	Kees Brinkman
	Casper Rokx
	Anna HE Roukens
**Additional Information:**	

Powered by Editorial Manager® and ProduXion Manager® from Aries Systems Corporation

Question	Response
**Financial Disclosure**<br><br>Enter a financial disclosure statement that describes the sources of funding for the work included in this submission. Review the submission guidelines for detailed requirements. View published research articles from *PLOS ONE* for specific examples.<br><br>This statement is required for submission and **will appear in the published article** if the submission is accepted. Please make sure it is accurate.<br><br>**Funded studies**<br>Enter a statement with the following details:<br>• Initials of the authors who received each award<br>• Grant numbers awarded to each author<br>• The full name of each funder<br>• URL of each funder website<br>• Did the sponsors or funders play any role in the study design, data collection and analysis, decision to publish, or preparation of the manuscript?<br><br>Did you receive funding for this work?	Yes
Please add funding details.<br>as follow-up to "**Financial Disclosure**<br><br>Enter a financial disclosure statement that describes the sources of funding for the work included in this submission. Review the submission guidelines for detailed requirements. View published research articles from *PLOS ONE* for specific examples.<br><br>This statement is required for submission and **will appear in the published article** if the submission is accepted. Please make sure it is accurate.	This trial was funded by the Dutch Organization for Health Research and Development (ZonMw) [grant number 10430072010008].

**Funded studies**<br>Enter a statement with the following details:<br>• Initials of the authors who received each award<br>• Grant numbers awarded to each author<br>• The full name of each funder<br>• URL of each funder website<br>• Did the sponsors or funders play any role in the study design, data collection and analysis, decision to publish, or preparation of the manuscript?<br><br>Did you receive funding for this work?"	
Please select the country of your main research funder (please select carefully as in some cases this is used in fee calculation).<br>as follow-up to "**Financial Disclosure**<br><br>Enter a financial disclosure statement that describes the sources of funding for the work included in this submission. Review the submission guidelines for detailed requirements. View published research articles from *PLOS ONE* for specific examples.<br><br>This statement is required for submission and **will appear in the published article** if the submission is accepted. Please make sure it is accurate.<br><br>**Funded studies**<br>Enter a statement with the following details:<br>• Initials of the authors who received each award<br>• Grant numbers awarded to each author<br>• The full name of each funder<br>• URL of each funder website<br>• Did the sponsors or funders play any role in the study design, data collection and analysis, decision to publish, or preparation of the manuscript?<br><br>Did you receive funding for this work?"	NETHERLANDS - NL
**Competing Interests**	RDdV is supported by the Health~Holland grant EMCLHS20017, co-funded by the

Use the instructions below to enter a competing interest statement for this submission. On behalf of all authors, disclose any competing interests that could be perceived to bias this work—acknowledging all financial support and any other relevant financial or non-financial competing interests.<br><br>This statement is required for submission and **will appear in the published article** if the submission is accepted. Please make sure it is accurate and that any funding sources listed in your Funding Information later in the submission form are also declared in your Financial Disclosure statement.<br><br>View published research articles from *PLOS ONE* for specific examples.<br><br>**NO authors have competing interests**<br><br>Enter: *The authors have declared that no competing interests exist.*<br><br>**Authors with competing interests**<br><br>Enter competing interest details beginning with this statement:<br><br>*I have read the journal's policy and the authors of this manuscript have the following competing interests: [insert competing interests here]*<br><br>* typeset	PPP Allowance made available by the Health~Holland, Top Sector Life Sciences & Health, to stimulate public–private partnerships, and is listed as an inventor of the fusion inhibitory lipopeptide [SARSHRC-PEG4]2-chol in a provisional patent application. KSH has received support for attending meetings and travel from Gilead. BJAR declares the receipt of research grants from Gilead and MSD and honoraria for advisory boards from AstraZeneca, Roche, Gilead, and F2G. KB has received research and educational grants from ViiV and Gilead, as well as consulting fees for advisory boards from ViiV, Gilead, MSD, and AstraZeneca. CR has received research grants from ViiV, Gilead, ZonMW, AIDSfonds, Erasmus MC, and Health~Holland, and honoraria for advisory boards from Gilead and ViiV. AR has received grants from the Bill and Melinda Gates Foundation and the Leids Universitair Fonds, participated on the board of an investor-initiated clinical trial on convalescent plasma for COVID-19, and is the chief editor of the Dutch Journal of Infectious Diseases and a member of the European Medicines Agency expert group on vaccines. All other authors declare no competing interests.
**Ethics Statement**<br><br>Enter an ethics statement for this submission. This statement is required if the study involved:<br><br>• Human participants<br>• Human specimens or tissue<br>• Vertebrate animals or cephalopods<br>• Vertebrate embryos or tissues	Human subject research was approved by the Medical Research Ethics Committee United

• Field research<br><br>Write "N/A" if the submission does not require an ethics statement.<br><br>General guidance is provided below. Consult the submission guidelines for detailed instructions. **Make sure that all information entered here is included in the Methods section of the manuscript.**	

**Format for specific study types**<br><br>**Human Subject Research (involving human participants and/or tissue)**<br>• Give the name of the institutional review board or ethics committee that approved the study<br>• Include the approval number and/or a statement indicating approval of this research<br>• Indicate the form of consent obtained (written/oral) or the reason that consent was not obtained (e.g. the data were analyzed anonymously)<br><br>**Animal Research (involving vertebrate animals, embryos or tissues)**<br>• Provide the name of the Institutional Animal Care and Use Committee (IACUC) or other relevant ethics board that reviewed the study protocol, and indicate whether they approved this research or granted a formal waiver of ethical approval<br>• Include an approval number if one was obtained<br>• If the study involved *non-human primates*, add *additional details* about animal welfare and steps taken to ameliorate suffering<br>• If anesthesia, euthanasia, or any kind of animal sacrifice is part of the study, include briefly which substances and/or methods were applied<br><br>**Field Research**<br><br>Include the following details if this study involves the collection of plant, animal, or other materials from a natural setting:<br>• Field permit number<br>• Name of the institution or relevant body that granted permission	
**Data Availability**<br><br>Authors are required to make all data underlying the findings described fully available, without restriction, and from the time of publication. PLOS allows rare exceptions to address legal and ethical concerns. See the PLOS Data Policy and FAQ for detailed information.	No - some restrictions will apply

Powered by Editorial Manager® and ProduXion Manager® from Aries Systems Corporation

A Data Availability Statement describing where the data can be found is required at submission. Your answers to this question constitute the Data Availability Statement and **will be published in the article**, if accepted.<br><br>**Important:** Stating 'data available on request from the author' is not sufficient. If your data are only available upon request, select 'No' for the first question and explain your exceptional situation in the text box.<br><br>Do the authors confirm that all data underlying the findings described in their manuscript are fully available without restriction?	
**Describe where the data may be found in full sentences. If you are copying our sample text, replace any instances of XXX with the appropriate details.**<br><br>• If the data are **held or will be held in a public repository**, include URLs, accession numbers or DOIs. If this information will only be available after acceptance, indicate this by ticking the box below. For example: *All XXX files are available from the XXX database (accession number(s) XXX, XXX.).*<br>• If the data are all contained **within the manuscript and/or Supporting Information files**, enter the following: *All relevant data are within the manuscript and its Supporting Information files.*<br>• If neither of these applies but you are able to provide **details of access elsewhere**, with or without limitations, please do so. For example:<br><br>*Data cannot be shared publicly because of [XXX]. Data are available from the XXX Institutional Data Access / Ethics Committee (contact via XXX) for researchers who meet the criteria for access to confidential data.*<br><br>*The data underlying the results presented in the study are available from (include the name of the third party*	Data will be available upon request

and contact information or URL).<br>• This text is appropriate if the data are owned by a third party and authors do not have permission to share the data.<br><br>* typeset	
Additional data availability information:	Tick here if the URLs/accession numbers/DOIs will be available only after acceptance of the manuscript for publication so that we can ensure their inclusion before publication.

Powered by Editorial Manager® and ProduXion Manager® from Aries Systems Corporation

Manuscript

Click here to access/download;Manuscript;Manuscript COVIH-LONG study_PLOS ONE.docx

Longitudinal assessment of COVID-19 vaccine immunogenicity in people with HIV stratified by CD4+ T-cell count in the Netherlands: a two-year follow-up study

Short Title: Two-year follow-up of COVID-19 vaccine immunogenicity in people with HIV stratified by CD4+ T-cell count

Marlou J. Jongkees[1] MD, Susanne Bogers[2] MSc, Rory D. de Vries[2] PhD, Corine H. GeurtsvanKessel[2] MD PhD, Pedro Miranda Afonso[3,4] MSc, Kathryn S. Hensley[1] MD, Bart J.A. Rijnders[1] MD PhD, Kees Brinkman[5] MD PhD, Casper Rokx[1¶] MD PhD, Anna H.E. Roukens[6*¶] MD PhD

[1] Department of Internal Medicine, Section Infectious Diseases, and Department of Medical Microbiology and Infectious Diseases, Erasmus University Medical Centre, Rotterdam, the Netherlands

[2] Department of Viroscience, Erasmus University Medical Centre, Rotterdam, the Netherlands

[3] Department of Biostatistics, Erasmus University Medical Centre, Rotterdam, the Netherlands

[4] Department of Epidemiology, Erasmus University Medical Centre, Rotterdam, the Netherlands

[5] Department of Internal Medicine and Infectious Diseases, OLVG Hospital, Amsterdam, the Netherlands

[6] Department of Infectious Diseases, Leiden University Medical Centre, Leiden, the Netherlands

[*] Corresponding author

E-mail: A.H.E.Roukens@lumc.nl (AHER)

[¶] These authors contributed equally to this work.

Abstract

Background: Guidelines for COVID-19 additional vaccination strategies for PWH are inconsistent worldwide, with some recommending an annual vaccination for all PWH, while others restrict this to PWH with a CD4+ T-cell count <200 cells per µL.

Methods: We conducted a prospective cohort study in 448 adult PWH. The primary outcome was the SARS-CoV-2 spike (S1)-specific antibody level at 1, 6, 12, 18, and 24 months after completing a primary COVID-19 vaccination series. We compared the antibody kinetics over two years between PWH with a baseline CD4+ T-cell count <200 (n=16) vs. ≥200 (n=432) with a mixed-effects model. Secondary outcomes included variables associated with the kinetics of S1-specific antibody levels and the incidence of breakthrough infections.

Results: The median most recent CD4+ T-cell count was 140 (IQR 80–165) in the <200 group, and 688 (IQR 520–899) in the ≥200 group at the time of primary vaccination. S1-specific antibodies were lower in PWH with a CD4+ T-cell count <200 vs. ≥200 during the two-year follow-up, with predicted S1-specific antibody levels of 514 (95% CI 456–578) vs. 2758 (95% CI 1488–5110) BAU/mL at 12 months ($p < 0.00001$) and 839 (95% CI 732–959) vs. 3505 (95% CI 1712–7175) BAU/mL at 24 months ($p = 0.0001$). The overall incidence of SARS-CoV-2 infections was 55% and comparable between groups. A CD4+ T-cell count <200, higher age, and a vector-based primary vaccination regimen were negatively associated with S1-specific antibody levels over time.

Conclusion: Long-term humoral responses were lower in PWH with a CD4+ T-cell count <200 compared to those with a CD4+ T-cell count ≥200. National COVID-19 vaccine guidelines recommending booster vaccines for all PWH, should therefore specifically emphasize the need for booster vaccines in those with a CD4+ T-cell count <200 cells per µL.

Trial registration: The trial was registered on the International Clinical Trials Platform (registration number: EUCTR2021-001054-57-N).

Keywords: CD4 Count; Durability; Follow-Up; SARS-CoV-2 Antibodies; COVID-19 Vaccine in PWH

Introduction

Since the beginning of the pandemic, higher coronavirus disease 2019 (COVID-19) mortality has been observed in immunocompromised individuals, including people with HIV (PWH) [1]. Although disease severity has decreased with the emergence of the Omicron variant, immunocompromised individuals remain at an increased risk of severe COVID-19 [2]. During the Omicron era, a higher risk of hospitalization due to COVID-19 has been reported in PWH compared to HIV-negative individuals [3].

Short-term immune responses following a primary COVID-19 vaccination regimen were lower in PWH compared to HIV-negative individuals [4–6], particularly in those with a CD4+ T-cell count <200 cells per µL. Larger studies indicate that this association between CD4+ T-cell count and antibody response persisted after booster vaccination [7–9]. An additional mRNA-1273 vaccination was highly effective in seroconverting PWH with a non-response after the primary vaccination series [10], and both monovalent and bivalent booster vaccinations improved neutralizing antibody responses in PWH [11–13].

Currently, COVID-19 vaccination studies in PWH do not exceed one year of follow-up. In addition to the lack of data on the durability of the immune response in PWH, risk factors for accelerated waning in PWH have not been assessed. For other vaccines, such as yellow fever and hepatitis B, a shorter duration of protection has been reported in PWH compared to HIV-negative individuals [14–16], with a lower CD4+ T-cell count associated with lower levels of neutralizing antibodies.

Understanding long-term immune responses is important, as the need for yearly COVID-19 booster vaccines for at-risk groups is currently being discussed worldwide. We hypothesize that PWH with a low CD4+ T-cell count exhibit lower spike (S)-specific antibody levels over time compared to PWH with a normal CD4+ T-cell count. Therefore, this study aimed to evaluate the durability of S-specific antibodies over a two-year period following COVID-19 vaccination among PWH.

Methods

Study design and participants

We performed a two-year follow-up of our nationwide prospective observational COVID-19 vaccination study among PWH in the Netherlands (COVIH) [6]. In this follow-up study, participants from the three COVIH coordinating HIV treatment centres (Erasmus Medical Centre, Leiden University Medical Centre, and OLVG Hospital) were monitored longitudinally for two years after completing the primary COVID-19 vaccination series. The inclusion criteria were a minimum age of 18 years and confirmed HIV infection. PWH were stratified into two subgroups based on their most recent CD4+ T-cell count (CD4+ T-cell count <200 vs. ≥200 cells per µL) prior to the primary vaccination series.

This study adhered to the Strengthening the Reporting of Observational Studies in Epidemiology (STROBE) guidelines to ensure comprehensive reporting of the data.

Clinical procedures

Between 22 February and 7 September 2021, 432 participants were included in the three HIV treatment centres. Participants were followed in routine care for two years after the primary vaccination series. To increase the number of participants with a CD4+ T-cell count <500 cells per µL, 16 additional participants were included retrospectively between 13 October 2023 and 11 April 2024, of whom six participants had a CD4+ T-cell count <200 cells per µL. Participants received two doses of BNT162b2, mRNA-1273, or ChAdOx1-S, or one dose of Ad26.COV2.S between January and August 2021 according to manufacturer's regulations as part of the Dutch COVID-19 vaccination campaign. All booster vaccinations administered were mRNA-based, including mRNA-1273, BNT162b2, mRNA-1273.214, BNT162b2 Omicron BA.1, mRNA-1273.222, BNT162b2 Omicron BA.5, mRNA-1273.815, and BNT162b2 Omicron XBB.1.5. Spike protein S1 subunit (S1)-specific antibodies were measured in blood samples taken during half-yearly HIV check-ups. Clinical data were collected in an electronic case

record file and included the following: year of birth, sex assigned at birth, current use of combination antiretroviral therapy (cART), most recent plasma HIV-RNA load (copies per mL), most recent CD4+ T-cell count (cells per µL) at the time of the primary vaccination series, CD4+ T-cell counts measured during the subsequent two years (cells per µL), nadir CD4+ T-cell count (cells per µL), comorbidities, co-medications, previous COVID-19 vaccinations, and history of self-reported breakthrough severe acute respiratory syndrome coronavirus 2 (SARS-CoV-2) infections confirmed by positive PCR or rapid antigen tests. Dates and types of previous COVID-19 vaccinations, as well as dates of intercurrent SARS-CoV-2 infections, including the test type, whether PCR or rapid antigen test, and infection severity, were obtained through yearly online questionnaires and telephone surveys.

Laboratory procedures

The concentrations of IgG binding antibodies specific to the ancestral S1-subunit were measured using a validated quantitative chemiluminescence immunoassay, the LIAISON SARS-CoV-2 TrimericS IgG assay (DiaSorin, Saluggia, Italy), with a lower limit of quantification of 4.81 BAU/mL, at the Erasmus University Medical Centre, a WHO SARS-CoV-2 reference laboratory.

Outcomes

The primary outcome was the level of S1-specific antibodies in PWH at 1, 6, 12, 18, and 24 months after primary vaccination. The kinetics of S1-specific antibodies over two years were compared between PWH with a CD4+ T-cell count <200 versus ≥200 cells per µL. Secondary outcomes included HIV-related and HIV-unrelated variables associated with the kinetics of S1-specific antibodies, as well as the incidence of self-reported SARS-CoV-2 breakthrough infections, confirmed by a positive PCR or rapid antigen test.

Statistical analysis plan

No power calculation was performed for this predefined secondary endpoint of the COVIH study [6]. All participants from the three COVIH coordinating centres in the original trial were eligible to participate in the current study. Data were described using number (percentage) or median (interquartile range [IQR]). A linear mixed-effects model was used to compare the kinetics of S1-specific antibodies over the two-year period between PWH with a most recent CD4+ T-cell count <200 vs. ≥200 cells per µL at the time of the primary vaccination regimen. The association between HIV-related and HIV-unrelated variables and the kinetics of S1-specific antibodies was analysed using a second model that included the following fixed effects: quadratic effect of time since the primary vaccination series, sex, age, most recent CD4+ T-cell count at baseline (<200 vs. ≥200 cells per µL), nadir CD4+ T-cell count, type of SARS-CoV-2 primary vaccination series (mRNA vs. vector), S1-specific antibody levels measured 4–6 weeks after the primary vaccination series, and time since the last COVID-19 vaccination dose or last SARS-CoV-2 infection. A likelihood ratio test was used to compare the longitudinal evolution between PWH with a most recent CD4+ T-cell count <200 and those with ≥200 cells per µL. Comparison of the SARS-CoV-2-infection-free probability between the groups was performed using a log-rank test. The proportion of PWH with a second and third infection were compared between both groups using a Chi-squared test. For all analyses, S1-specific antibodies were log_{10}-transformed. P values < 0.05 were considered statistically significant. Undetectable S1-specific antibody responses (< 4.81 BAU/mL) were reported as 4.81 in the statistical analyses. Data were analysed using RStudio 4.3.2 and the nlme package (v. 3.1.162).

Ethics committee approval

The trial was conducted in accordance with the Declaration of Helsinki, Good Clinical Practice guidelines, and the Dutch Medical Research Involving Human Subjects Act (WMO). It was approved by the Medical Ethics Committees United Nieuwegein (MEC-U, reference 20.125) and registered on the International Clinical Trials Platform (EUCTR2021-001054-57-N). All participants provided written informed consent.

Role of funding source

The funder of the study had no role in the study design, data collection, data analysis, data interpretation, or writing of the report.

Results

Baseline characteristics

Of the 448 participants enrolled in the COVIH follow-up study, 16 participants had a most recent CD4+ T-cell count <200 cells per µL at the time of the primary vaccination series (3.6%) (Fig 1). Over the two-year follow-up period, nine of the 16 participants in the baseline CD4 <200 group showed an increase to ≥200 cells per µL, while three of the 432 participants in the baseline CD4 ≥200 group experienced a decrease to <200 cells per µL. Ten participants dropped out during follow-up: four died and six moved to another hospital. A total of 2142 samples were analysed, including 77 samples from participants in the baseline CD4 <200 group and 2065 from participants in the baseline CD4 ≥200 group. Overall, the questionnaire response rate was 90.0%.

Fig 1. Flowchart of included PWH in the two-year follow-up of the COVIH study. *n* denotes the number of unique individuals.

The cohort was predominantly male (87.1%), with a median age of 56 years (IQR 47–63) (Table 1). Participants in the baseline CD4 <200 group had a median most recent CD4+ T-cell count of 140 cells per µL (IQR 80–165) and a median nadir CD4+ T-cell count of 25 cells per µL (IQR 10–125). In contrast, participants in the baseline CD4 ≥200 group had a median most recent CD4+ T-cell count of 688 cells per µL (IQR 520–899) and a median nadir CD4+ cell count of 230 cells per µL (IQR 100–324). All participants were on combination antiretroviral therapy (cART), except for one elite controller. Almost all participants were virally suppressed, with 98.2% having a plasma HIV viral load <50 copies per mL at inclusion. Most participants had received BNT162b2 (77.5%) or ChAdOx1-S (15.0%) for their primary vaccination series, while a smaller proportion had received mRNA-1273 (6.2%) or Ad26.COV2.S (1.3%).

Table 1. Baseline characteristics of participants stratified by CD4+ T-cell count.

	**All PWH**<br>N = 448	**PWH with a CD4+ T-cell count <200 cells per µL**<br>N = 16	**PWH with a CD4+ T-cell count ≥200 cells per µL**<br>N = 432
**Age**, years[a]	56 (47–63)	55 (45–59)	56 (47–63)
**Male**	390 (87.1%)	13 (81.3%)	377 (87.3%)
**Female**	58 (12.9%)	3 (18.7%)	55 (12.7%)
**On cART**	447 (99.8%)	16 (100%)	431 (99.8%)
**Most recent plasma HIV viral load <50**, copies per mL	440 (98.2%)	14 (87.5%)	426 (98.6%)
**Most recent plasma HIV viral load <200**, copies per mL	446 (99.6%)	15 (93.8%)	431 (99.8%)
**Most recent CD4+ T-cell count,** cells per µL	675 (509–894)	140 (80–165)	688 (520–899)
**Nadir CD4+ T-cell count**, cells per µL	220 (84–320)	25 (10–125)	230 (100–324)
**COVID-19 vaccine type for primary vaccination series**			
BNT162b2	347 (77.5%)	13 (81.3%)	334 (77.3%)
mRNA-1273	28 (6.2%)	2 (12.5%)	26 (6.0%)
ChAdOx1-S	67 (15.0%)	1 (6.2%)	66 (15.3%)

Ad26.COV2.S	6 (1.3%)	0	6 (1.4%)
**COVID-19 vaccinations received**[b]			
Primary series only	31 (7.7%)	2 (13.3%)	29 (7.5%)
Primary series + 1 booster	103 (25.6%)	4 (26.7%)	99 (25.5%)
Primary series + 2 boosters	139 (34.5%)	4 (26.7%)	136 (35.1%)
Primary series + 3 boosters	121 (30.0%)	5 (33.3%)	115 (29.6%)
Primary series + 4 boosters	9 (2.2%)	0	9 (2.3%)
**History of at least one SARS-CoV-2 infection**[b,c]			
Yes	225 (54.9%)	10 (62.5%)	215 (54.6%)
No	185 (45.1%)	6 (37.5%)	179 (45.4%)
**Dominant variant in the Netherlands at the time of first reported SARS-CoV-2 infection** [17]			
Alpha variant[b]	7 (3.1%)	1 (10.0%)	6 (2.8%)
Delta variant[b]	24 (10.7%)	0	24 (11.2%)
Omicron variant[b]	194 (86.2%)	9 (90.0%)	185 (86.0%)
**History of a second SARS-CoV-2 infection**[b]	37 (9.0%)	2 (12.5%)	35 (8.9%)

**History of a third SARS-CoV-2 infection**[b]	5 (1.2%)	1 (6.3%)	4 (1.0%)
**S1-specific antibodies 4–6 weeks after primary vaccination**, BAU per mL	1108 (967–1270)	65 (188–236)	1210 (1065–1374)

Data are n (%), median (IQR), or geometric mean (95% CI). Abbreviations: BAU, binding antibody units; cART, combination antiretroviral therapy; HIV, human immunodeficiency virus; IQR, interquartile range; S, spike.

[a] At baseline (defined as 1 January 2021).

[b] Missing data were excluded from denominators: COVID-19 vaccinations received: 45 (10.0%) overall, 1 (6.3%) in the <200 group, 44 (10.2%) in the ≥200 group; History of SARS-CoV-2 infection: 38 (8.5%) overall, none in the baseline CD4 <200 group, 38 (8.8%) in the baseline CD4 ≥200 group.

[c] Confirmed by a self-reported positive polymerase chain reaction test or rapid antigen test.

Kinetics of antibody responses

S1-specific antibody levels were lower in PWH with a baseline CD4+ T-cell count <200 cells per µL compared to those with a baseline CD4+ T-cell count ≥200 cells per µL in the two years following the primary vaccination series (p<0.00001) (Fig 2). Predicted S1-specific antibodies levels were lower in the baseline CD4 <200 group compared to the baseline CD4 ≥200 group at the following time points: 168 (95% CI 151–187) vs. 1504 (95% CI 858–2636) BAU/mL at month 6 (p < 0.00001), 514 (95% CI 456–578) vs. 2758 (95% CI 1488–5110) BAU/mL at month 12 (p < 0.00001), 879 (95% CI 784–987) vs. 3660 (95% CI 2008–6671) BAU/mL at month 18 (p < 0.00001) and 839 (95% CI 732–959) vs. 3505 (95% CI 1712–7175) BAU/mL at month 24 (p = 0.0001), respectively. The median number of booster vaccine doses received after completing the primary vaccination series was 2 (IQR 1–3) in both groups. The type of COVID-19 booster vaccines BNT162b2 vs. mRNA-1273 was comparable between the groups.

Fig 2. SARS-CoV-2 spike (S1)-specific antibody concentration (BAU/mL) in PWH stratified by CD4+ T-cell count over time (days) from immediately prior primary vaccination (defined as day 0). Comparison between PWH with a CD4+ T-cell count <200 cells per µL (n=16; red) and PWH with a CD4+ T-cell count ≥200 cells per µL (n=432; blue) was performed using the likelihood ratio test. The solid lines represent the mixed-effects regression of the $\log_{10}$ S1-specific antibody levels per baseline CD4 group over time, with the dotted lines representing the 95% confidence interval. Abbreviations: BAU, binding antibody unit; LLoD, lower limit of detection; S, spike.

Variables associated with the kinetics of antibody responses

A CD4+ T-cell count <200 cells per µL (p < 0.00001), older age (p = 0.001), a vector-based primary vaccination regimen (p < 0.00001), and increased time since the last COVID-19 vaccination dose or last SARS-CoV-2 infection (p < 0.00001) were associated with lower S1-specific antibody levels over time (S2 Table). Higher S1-specific antibody levels one month after the primary vaccination series (p <

0.00001) was positively associated with S1-specific antibody levels over time. No association was found for sex or nadir CD4+ T-cell count.

Incidence of SARS-CoV-2 infection

No significant difference was observed in the probability of remaining free of the first SARS-CoV-2 infection between PWH with a CD4+ T-cell count <200 cells per µL (46.7% (95%CI 27.2–80.2)) and those with a CD4+ T-cell count ≥200 cells per µL (45.0% (95%CI 40.4–50.2)) during the two-year follow-up ($p = 0.83$). Fig 3 shows the corresponding cumulative risk functions. A second or third SARS-CoV-2 infection was reported in 12.5% and 6.3% of participants in the baseline CD4 <200 group compared to 8.9% and 1.0% in the baseline CD4 ≥200 group ($p = 0.89$ and $p = 0.44$, respectively).

Fig 3. Probability of remaining free of the first SARS-CoV-2 infection during the two-year follow-up in PWH stratified by CD4+ T-cell count. Comparison between PWH with a CD4+ T-cell count <200 (n = 16; red) and PWH with a CD4+ T-cell count ≥200 (n = 432; blue) was performed by a log-rank test. Data on SARS-CoV-2 infection history was missing from 38 (8.5%) participants, of whom none in the baseline CD4 <200 group and 38 (8.8%) in the baseline CD4 ≥200 group.

SARS-CoV-2 infection severity

Four participants (0.9%) required treatment for a SARS-CoV-2 infection (S3 Table). Of these, one participant – a 61-year-old male – was admitted to the intensive care unit. His most recent CD4+ T-cell count was 316 cells per µL, and his nadir CD4+ T-cell count was 50 cells per µL. Two other participants were hospitalized due to a SARS-CoV-2 infection: an 80-year-old male with a most recent CD4+ T-cell count of 1210 cells per µL, and a 64-year-old male with a most recent CD4+ T-cell count of 360 cells per µL. Lastly, a 75-year-old male with a most recent CD4+ T-cell count of 700 cells per µL received nirmatrelvir/ritonavir treatment.

Discussion

Our two year follow-up shows that PWH with a CD4+ T-cell count <200 cells per µL had consistently lower antibody levels after COVID-19 vaccination compared to PWH with a CD4+ T-cell count ≥200 cells per µL. The comparable SARS-CoV-2 infection rates and received booster doses between the groups, suggest that the difference in S1-specific antibodies is not explained by these factors.

Despite the comparatively lower antibody levels in PWH with a CD4+ T-cell count <200 cells per µL, from month 6 onwards, S1-specific antibodies in this group remained above 300 BAU/mL – a threshold previously correlated with the presence of neutralizing antibodies against the Delta variant [18]. As the currently circulating variants from the different Omicron sublineages are antigenically even more distinct [19], breakthrough infections are expected, and were indeed observed in our cohort. An association between higher predicted spike-specific antibody levels and a lower risk of Omicron infection in both PWH and HIV-negative individuals has been demonstrated [20]. Since COVID-19 caused by SARS-CoV-2 from the Omicron sublineages is less severe, either by viral mutations or blunting by pre-existing immunity, we propose to continue the strategy of administering a yearly updated booster vaccine to PWH with a CD4+ T-cell count <200 cells per µL, particularly if the case of discontinuing the yearly booster vaccine for all PWH. Given the potential for new variants to emerge, it remains important to reassess immune responses and vaccination strategies periodically, especially in individuals with advanced HIV infection.

To our knowledge, this is the first longitudinal study in PWH extending beyond one year of follow-up after COVID-19 vaccination. A key strength of our study is the large cohort size and particularly the inclusion of a sufficient number of PWH with a CD4+ T-cell count <200 cells per µL. Another strength is the comprehensive follow-up starting from before the first COVID-19 vaccine dose, with half-yearly blood sampling and yearly documentations of booster vaccine doses as well as SARS-CoV-2 breakthrough infections.

Our study has some limitations. In particular, we did not assess cellular immune responses or functional antibody responses, although S1-specific antibodies are known to correlate well with neutralizing activity [18,21]. Additionally, almost all participants were on combination antiretroviral therapy, which limits the generalizability of our findings to regions with limited access to HIV suppressive therapy. While previous studies have shown comparable humoral responses in well-treated PWH with normal CD4+ T-cell counts and HIV-negative controls up to one year of follow-up, the absence of a control group without HIV in this study should be noted. Lastly, the incidence of self-reported SARS-CoV-2 infections and prior vaccinations were collected through online questionnaires and telephone surveys, introducing the potential for recall bias. However, data on booster vaccinations were registered by the Dutch National Institute for Public Health and the Environment, minimizing recall bias in vaccination history.

In conclusion, PWH with a CD4+ T-cell count <200 cells per µL had lower humoral responses over the two years following the primary COVID-19 vaccination series. National COVID-19 vaccine guidelines recommending booster vaccines for all PWH should therefore specifically emphasize the need for booster vaccines in those with a CD4+ T-cell count <200 cells per µL.

Acknowledgments

Foremost, we thank all participants of the COVIH study. We also thank Aarti Soenessardien and Nieven Zhu for their valuable assistance in data collection.

References

1. Leston M, Elson W, Ordóñez-Mena JM, et al. Disparities in COVID-19 mortality amongst the immunosuppressed: A systematic review and meta-analysis for enhanced disease surveillance. J Infect 2024; 88(3): 106110.

2. Evans RA, Dube S, Lu Y, et al. Impact of COVID-19 on immunocompromised populations during the Omicron era: insights from the observational population-based INFORM study. Lancet Reg Health Eur 2023; 35: 100747.

3. Killander Möller I, Hedberg P, Wagner P, et al. Risk of COVID-19 hospitalisation by HIV-status and SARS-CoV-2 vaccination status during pre- and post-Omicron era in a national register-based cohort study in Sweden. Infect Dis (Lond) 2024: 1–14.

4. Søndergaard MH, Thavarajah JJ, Churchill Henson H, Wejse CM. SARS-CoV-2 vaccine immunogenicity for people living with HIV: A systematic review and meta-analysis. HIV Med 2024; 25(1): 16–37.

5. Griffin DWJ, Pai Mangalore R, Hoy JF, McMahon JH. Immunogenicity, effectiveness, and safety of SARS-CoV-2 vaccination in people with HIV. Aids 2023; 37(9): 1345–60.

6. Hensley KS, Jongkees MJ, Geers D, et al. Immunogenicity and reactogenicity of SARS-CoV-2 vaccines in people living with HIV in the Netherlands: A nationwide prospective cohort study. PLoS Med 2022; 19(10): e1003979.

7. Vergori A, Tavelli A, Matusali G, et al. SARS-CoV-2 mRNA Vaccine Response in People Living with HIV According to CD4 Count and CD4/CD8 Ratio. Vaccines (Basel) 2023; 11(11).

8. Costiniuk CT, Singer J, Lee T, et al. COVID-19 vaccine immunogenicity in people with HIV. Aids 2023; 37(1): F1–F10.

9. Tau L, Turner D, Adler A, et al. SARS-CoV-2 Humoral and Cellular Immune Responses of Patients With HIV After Vaccination With BNT162b2 mRNA COVID-19 Vaccine in the Tel-Aviv Medical Center. Open Forum Infect Dis 2022; 9(4): ofac089.

10. Jongkees MJ, Geers D, Hensley KS, et al. Immunogenicity of an Additional mRNA-1273 SARS-CoV-2 Vaccination in People With HIV With Hyporesponse After Primary Vaccination. J Infect Dis 2023; 227(5): 651–62.

11. Cheng MQ, Li R, Weng ZY, Song G. Immunogenicity and effectiveness of COVID-19 booster vaccination among people living with HIV: a systematic review and meta-analysis. Front Med (Lausanne) 2023; 10: 1275843.

12. Vergori A, Matusali G, Lepri AC, et al. Neutralizing activity and T-cell response after bivalent fifth dose of messenger RNA vaccine in people living with HIV. Int J Infect Dis 2023; 134: 195–9.

13. Jongkees MJ, Tan NH, Geers D, et al. Immunogenicity of a bivalent BA.1 COVID-19 booster vaccine in people with HIV in the Netherlands. Aids 2024; 38(9): 1355–65.

14. Motta E, Camacho LAB, Cunha M, et al. Immunogenicity and reactogenicity of yellow fever vaccine in people with HIV. Aids 2023; 37(15): 2319–29.

15. Veit O, Niedrig M, Chapuis-Taillard C, et al. Immunogenicity and safety of yellow fever vaccination for 102 HIV-infected patients. Clin Infect Dis 2009; 48(5): 659–66.

16. Feng YL, Chang Y, Shi J, et al. Immunization effect and persistence of hepatitis B vaccine in HIV-infected patients with different CD4(+)T cell levels. Zhonghua Liu Xing Bing Xue Za Zhi 2021; 42(9): 1559–65.

17. Dutch National Institute for Public Health and the Environment. Varianten van het coronavirus SARS-CoV-2. https://www.rivm.nl/corona/actueel/virusvarianten. Accessed October 1, 2024.

18. Prins MLM, Roozen GVT, Pothast CR, et al. Immunogenicity and reactogenicity of intradermal mRNA-1273 SARS-CoV-2 vaccination: a non-inferiority, randomized-controlled trial. NPJ Vaccines 2024; 9(1): 1.

19. Mykytyn AZ, Rosu ME, Kok A, et al. Antigenic mapping of emerging SARS-CoV-2 omicron variants BM.1.1.1, BQ.1.1, and XBB.1. Lancet Microbe 2023; 4(5): e294–e5.

20. Verburgh ML, Boyd A, Schim van der Loeff MF, et al. Similar Limited Protection Against Severe Acute Respiratory Syndrome Coronavirus 2 Omicron Infection in Vaccinated Individuals With HIV and Comparable Controls. Open Forum Infect Dis 2024; 11(7): ofae380.

21. Sun K, Bhiman JN, Tempia S, et al. SARS-CoV-2 correlates of protection from infection against variants of concern. Nat Med 2024. (Forthcoming)

Supporting information

S1 Table. Overview national guidelines on COVID-19 vaccination.

S2 Table. Linear mixed-effects model of variables associated with S1-specific antibody levels over time. The association between participant-related and vaccine-related variables on the magnitude of spike (S1)-specific antibodies overall, and how the association of these variables with S1-specific antibodies changes over linear and quadratic time, was evaluated by a linear mixed-effects model.

S3 Table. Characteristics of the four participants who received treatment for a SARS-CoV-2 infection.

Figure 1

Click here to access/download;Figure;Fig 1.pdf

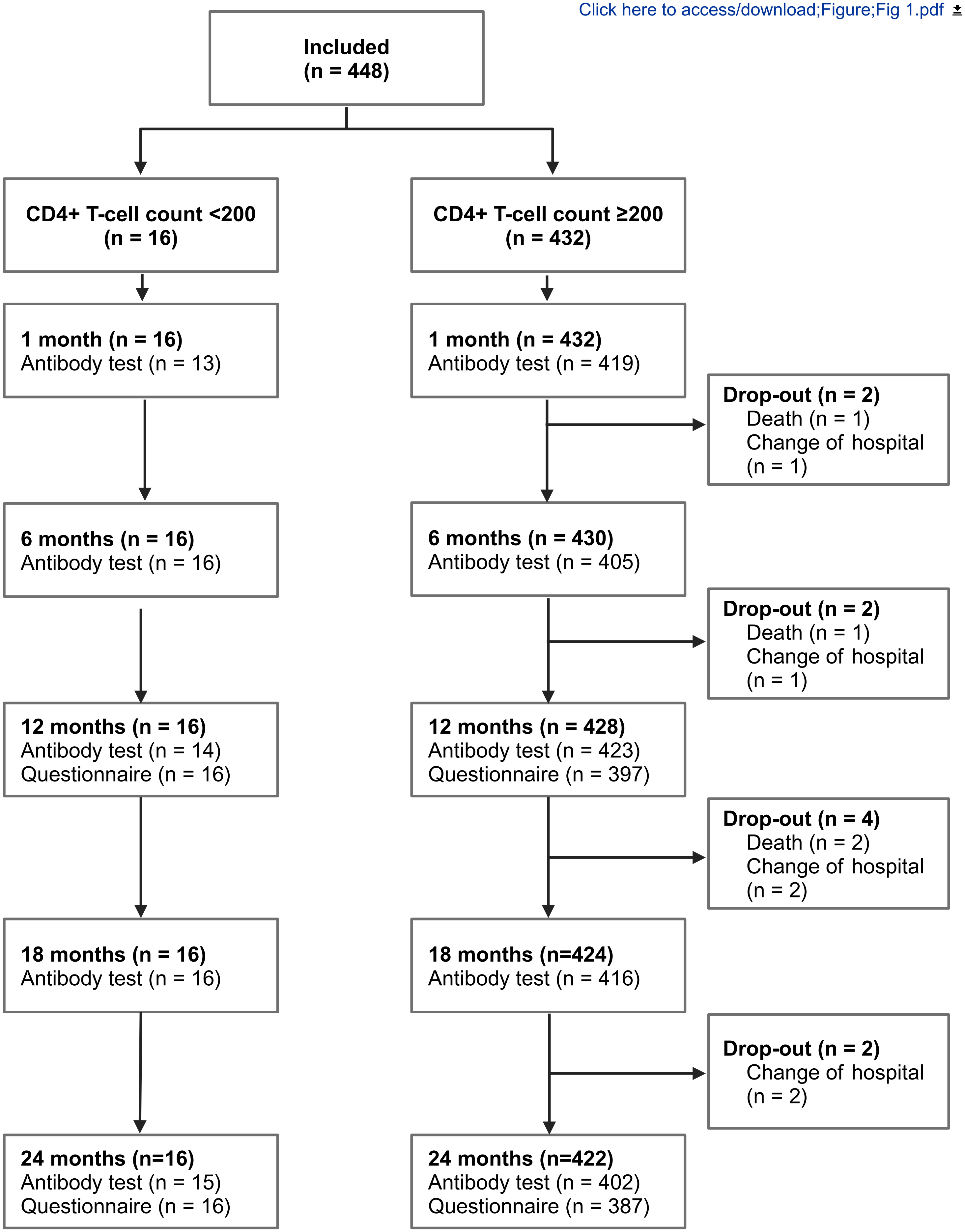

Figure 2

Click here to access/download;Figure;Fig 2.tiff

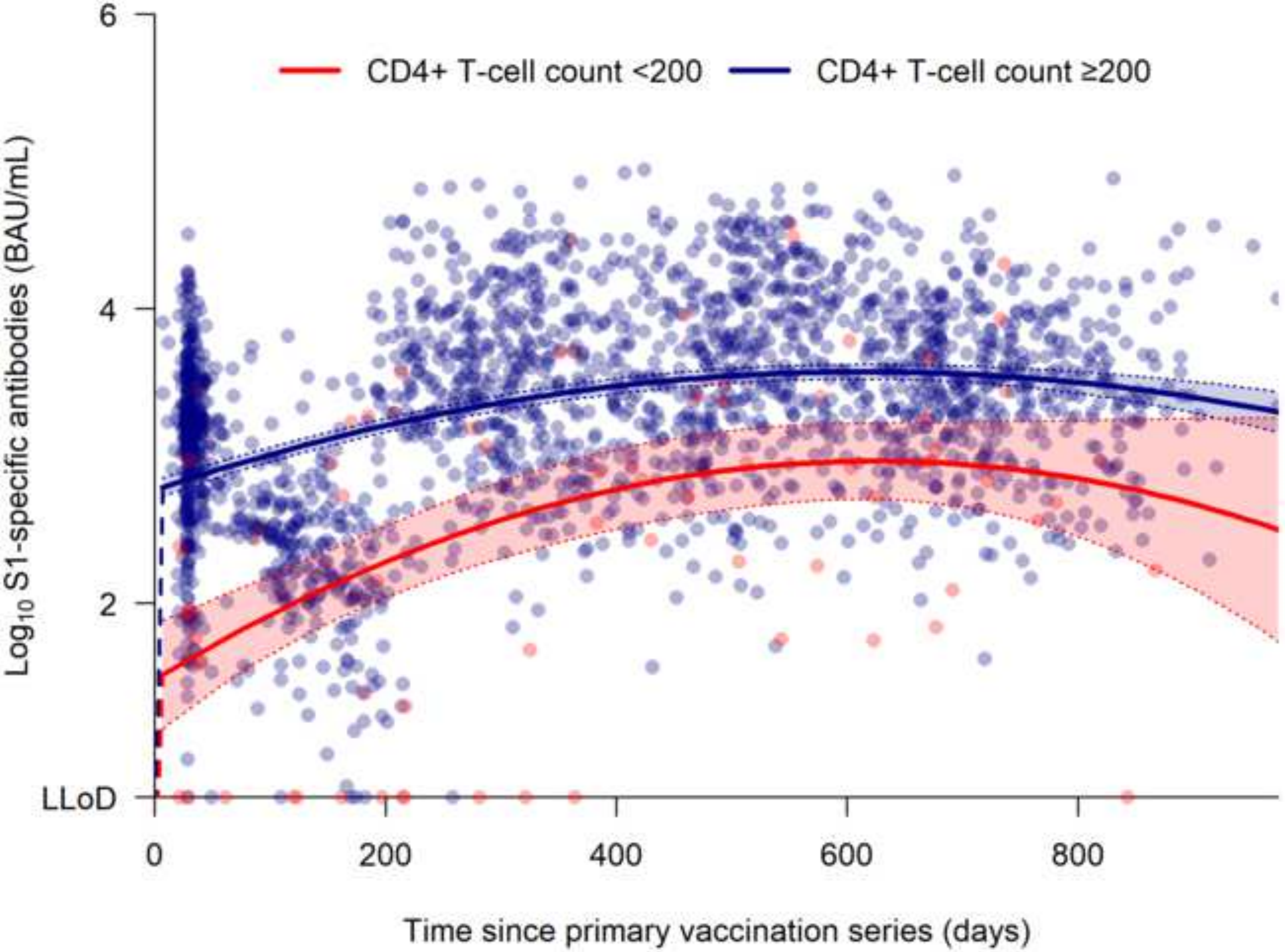

Figure 3

Click here to access/download;Figure;Fig 3.tiff

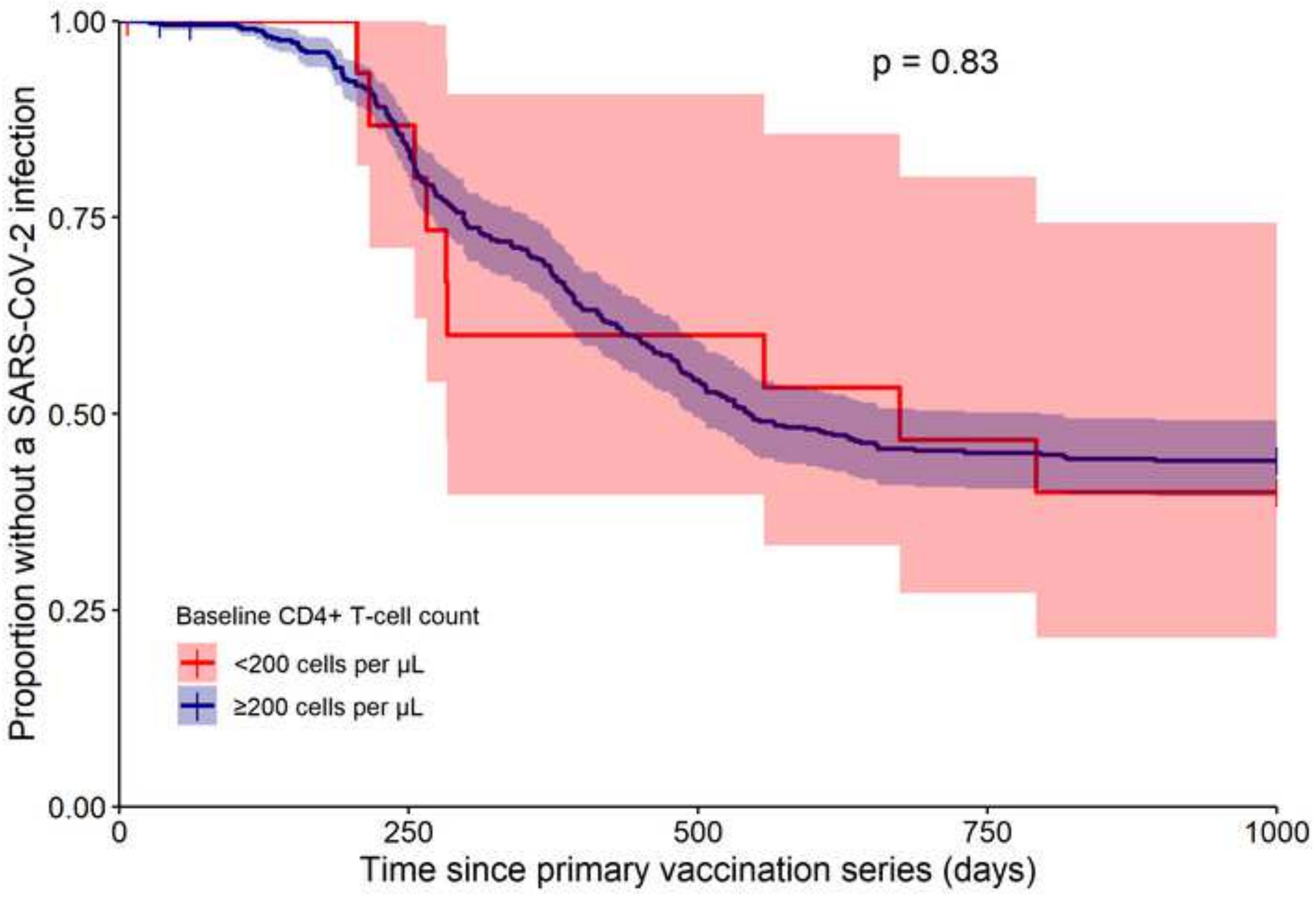

Click here to access/download
Supporting Information
S1 Table.docx

Click here to access/download
Supporting Information
S2 Table.docx

Click here to access/download
Supporting Information
S3 Table.docx
