# Supplementary material for: Longitudinal assessment of COVID-19 vaccine immunogenicity in people with HIV stratified by CD4+ T-cell count in the Netherlands: A two-year follow-up study
Source: PLoS One. 2025 May 19;20(5):e0323792. doi: 10.1371/journal.pone.0323792 (PMC12087993; doi:10.1371/journal.pone.0323792)
Supplement: Attachment — Submitted filename: Response to Reviewers.docx [file pone.0323792.s008.docx]

**Response to Reviewers**

1. Please ensure that your manuscript meets PLOS ONE's style requirements, including those for file naming. The PLOS ONE style templates can be found at 
[https://journals.plos.org/plosone/s/file?id=wjVg/PLOSOne_formatting_sample_main_body.pdf](https://eur01.safelinks.protection.outlook.com/?url=https%3A%2F%2Fjournals.plos.org%2Fplosone%2Fs%2Ffile%3Fid%3DwjVg%2FPLOSOne_formatting_sample_main_body.pdf&data=05%7C02%7Cm.jongkees%40erasmusmc.nl%7Ce925bc084456499b648608dd21a5c36b%7C526638ba6af34b0fa532a1a511f4ac80%7C0%7C0%7C638703718795486100%7CUnknown%7CTWFpbGZsb3d8eyJFbXB0eU1hcGkiOnRydWUsIlYiOiIwLjAuMDAwMCIsIlAiOiJXaW4zMiIsIkFOIjoiTWFpbCIsIldUIjoyfQ%3D%3D%7C0%7C%7C%7C&sdata=naujEPEbmAZamVIe6fzSFPZ30se%2FmwZR8dSHygnKwbM%3D&reserved=0) and 
[https://journals.plos.org/plosone/s/file?id=ba62/PLOSOne_formatting_sample_title_authors_affiliations.pdf](https://eur01.safelinks.protection.outlook.com/?url=https%3A%2F%2Fjournals.plos.org%2Fplosone%2Fs%2Ffile%3Fid%3Dba62%2FPLOSOne_formatting_sample_title_authors_affiliations.pdf&data=05%7C02%7Cm.jongkees%40erasmusmc.nl%7Ce925bc084456499b648608dd21a5c36b%7C526638ba6af34b0fa532a1a511f4ac80%7C0%7C0%7C638703718795502215%7CUnknown%7CTWFpbGZsb3d8eyJFbXB0eU1hcGkiOnRydWUsIlYiOiIwLjAuMDAwMCIsIlAiOiJXaW4zMiIsIkFOIjoiTWFpbCIsIldUIjoyfQ%3D%3D%7C0%7C%7C%7C&sdata=%2BKGAsHX%2FEcTs7HeGqWpW3C2i87CeLPty1JwfY5aTOrs%3D&reserved=0)

We have ensured that our manuscript complies with the style requirements, including those for file naming.

2. Thank you for stating the following financial disclosure: 
This trial was funded by the Dutch Organization for Health Research and Development (ZonMw) [grant number 10430072010008].

Please state what role the funders took in the study.  If the funders had no role, please state: ""The funders had no role in study design, data collection and analysis, decision to publish, or preparation of the manuscript."" 
If this statement is not correct you must amend it as needed. 
Please include this amended Role of Funder statement in your cover letter; we will change the online submission form on your behalf.

The following statement is correct: "The funders had no role in study design, data collection and analysis, decision to publish, or preparation of the manuscript. " We have added this Role of Funder statement in our cover letter.

3. Thank you for stating the following in the Competing Interests section: 
RDdV is supported by the Health~Holland grant EMCLHS20017, co-funded by the PPP Allowance made available by the Health~Holland, Top Sector Life Sciences & Health, to stimulate public–private partnerships, and is listed as an inventor of the fusion inhibitory lipopeptide [SARSHRC-PEG4]2-chol in a provisional patent application. KSH has received support for attending meetings and travel from Gilead. BJAR declares the receipt of research grants from Gilead and MSD and honoraria for advisory boards from AstraZeneca, Roche, Gilead, and F2G. KB has received research and educational grants from ViiV and Gilead, as well as consulting fees for advisory boards from ViiV, Gilead, MSD, and AstraZeneca. CR has received research grants from ViiV, Gilead, ZonMW, AIDSfonds, Erasmus MC, and Health~Holland, and honoraria for advisory boards from Gilead and ViiV. AR has received grants from the Bill and Melinda Gates Foundation and the Leids Universitair Fonds, participated on the board of an investor-initiated clinical trial on convalescent plasma for COVID-19, and is the chief editor of the Dutch Journal of Infectious Diseases and a member of the European Medicines Agency expert group on vaccines. All other authors declare no competing interests.

Please confirm that this does not alter your adherence to all PLOS ONE policies on sharing data and materials, by including the following statement: ""This does not alter our adherence to  ONE policies on sharing data and materials.” (as detailed online in our guide for authors [http://journals.plos.org/plosone/s/competing-interests](https://eur01.safelinks.protection.outlook.com/?url=http%3A%2F%2Fjournals.plos.org%2Fplosone%2Fs%2Fcompeting-interests&data=05%7C02%7Cm.jongkees%40erasmusmc.nl%7Ce925bc084456499b648608dd21a5c36b%7C526638ba6af34b0fa532a1a511f4ac80%7C0%7C0%7C638703718795518508%7CUnknown%7CTWFpbGZsb3d8eyJFbXB0eU1hcGkiOnRydWUsIlYiOiIwLjAuMDAwMCIsIlAiOiJXaW4zMiIsIkFOIjoiTWFpbCIsIldUIjoyfQ%3D%3D%7C0%7C%7C%7C&sdata=DagboHwgGJodPFS8857%2FD5DsSQ0KHB6iXFNl6FaQIsc%3D&reserved=0)).  If there are restrictions on sharing of data and/or materials, please state these. Please note that we cannot proceed with consideration of your article until this information has been declared.

Please include your updated Competing Interests statement in your cover letter; we will change the online submission form on your behalf.

The following statement "This does not alter our adherence to PLOS ONE policies on sharing data and materials.” has been added to the Competing Interests statement and we have rewritten the last sentence to "The other authors have declared that no competing interests exist.” Find the cover letter for the full updated Competing Interests statement.

4. Please include your full ethics statement in the ‘Methods’ section of your manuscript file. In your statement, please include the full name of the IRB or ethics committee who approved or waived your study, as well as whether or not you obtained informed written or verbal consent. If consent was waived for your study, please include this information in your statement as well.

The full ethics statement is included in the ‘Methods’ section, including the full name of the ethics committee and that all participants provided written informed consent.

5. In the online submission form, you indicated that data will be available upon request.

All PLOS journals now require all data underlying the findings described in their manuscript to be freely available to other researchers, either 1. In a public repository, 2. Within the manuscript itself, or 3. Uploaded as supplementary information.
This policy applies to all data except where public deposition would breach compliance with the protocol approved by your research ethics board. If your data cannot be made publicly available for ethical or legal reasons (e.g., public availability would compromise patient privacy), please explain your reasons on resubmission and your exemption request will be escalated for approval.

We chose to share data under restricted access, as we are advised to do by our datamanagers. The data are pseudonymised data from real patients and we want to protect these maximally, as participants singed the informed consent saying that they cannot be identified from the data. According to our datamanagers this cannot be assured for pseudonymised data, as the data include potentially identifying and sensitive patient information.

6. We note that you have indicated that there are restrictions to data sharing for this study. For studies involving human research participant data or other sensitive data, we encourage authors to share de-identified or anonymized data. However, when data cannot be publicly shared for ethical reasons, we allow authors to make their data sets available upon request. For information on unacceptable data access restrictions, please see [http://journals.plos.org/plosone/s/data-availability#loc-unacceptable-data-access-restrictions](https://eur01.safelinks.protection.outlook.com/?url=http%3A%2F%2Fjournals.plos.org%2Fplosone%2Fs%2Fdata-availability%23loc-unacceptable-data-access-restrictions&data=05%7C02%7Cm.jongkees%40erasmusmc.nl%7Ce925bc084456499b648608dd21a5c36b%7C526638ba6af34b0fa532a1a511f4ac80%7C0%7C0%7C638703718795534662%7CUnknown%7CTWFpbGZsb3d8eyJFbXB0eU1hcGkiOnRydWUsIlYiOiIwLjAuMDAwMCIsIlAiOiJXaW4zMiIsIkFOIjoiTWFpbCIsIldUIjoyfQ%3D%3D%7C0%7C%7C%7C&sdata=kTZLA6yxs%2F2bZ3e%2FOraNSgINlWGzu8dn6%2FaqZVhkd44%3D&reserved=0).

Before we proceed with your manuscript, please address the following prompts:

a) If there are ethical or legal restrictions on sharing a de-identified data set, please explain them in detail (e.g., data contain potentially identifying or sensitive patient information, data are owned by a third-party organization, etc.) and who has imposed them (e.g. a Research Ethics Committee or Institutional Review Board, etc.). Please also provide contact information for a data access committee, ethics committee, or other institutional body to which data requests may be sent.

b) If there are no restrictions, please upload the minimal anonymized data set necessary to replicate your study findings to a stable, public repository and provide us with the relevant URLs, DOIs, or accession numbers. Please see [http://www.bmj.com/content/340/bmj.c181.long](https://eur01.safelinks.protection.outlook.com/?url=http%3A%2F%2Fwww.bmj.com%2Fcontent%2F340%2Fbmj.c181.long&data=05%7C02%7Cm.jongkees%40erasmusmc.nl%7Ce925bc084456499b648608dd21a5c36b%7C526638ba6af34b0fa532a1a511f4ac80%7C0%7C0%7C638703718795550718%7CUnknown%7CTWFpbGZsb3d8eyJFbXB0eU1hcGkiOnRydWUsIlYiOiIwLjAuMDAwMCIsIlAiOiJXaW4zMiIsIkFOIjoiTWFpbCIsIldUIjoyfQ%3D%3D%7C0%7C%7C%7C&sdata=bGKtzhUdONstk4NlA1NOGuo4wIryJqeu50PyTFHQI%2Bs%3D&reserved=0) for guidelines on how to de-identify and prepare clinical data for publication. For a list of recommended repositories, please see [https://journals.plos.org/plosone/s/recommended-repositories](https://eur01.safelinks.protection.outlook.com/?url=https%3A%2F%2Fjournals.plos.org%2Fplosone%2Fs%2Frecommended-repositories&data=05%7C02%7Cm.jongkees%40erasmusmc.nl%7Ce925bc084456499b648608dd21a5c36b%7C526638ba6af34b0fa532a1a511f4ac80%7C0%7C0%7C638703718795566934%7CUnknown%7CTWFpbGZsb3d8eyJFbXB0eU1hcGkiOnRydWUsIlYiOiIwLjAuMDAwMCIsIlAiOiJXaW4zMiIsIkFOIjoiTWFpbCIsIldUIjoyfQ%3D%3D%7C0%7C%7C%7C&sdata=SxQMUQSZf6Z2BXTXEdg7Wi22UXih9N0aAuRky3THByw%3D&reserved=0). You also have the option of uploading the data as Supporting Information files, but we would recommend depositing data directly to a data repository if possible.

Please update your Data Availability statement in the submission form accordingly.

We have updated our Data Availability statement in the submission form.

7. Please remove all personal information, ensure that the data shared are in accordance with participant consent, and re-upload a fully anonymized data set.

See our response to comment 6.

Note: spreadsheet columns with personal information must be removed and not hidden as all hidden columns will appear in the published file.

Additional guidance on preparing raw data for publication can be found in our Data Policy ([https://journals.plos.org/plosone/s/data-availability#loc-human-research-participant-data-and-other-sensitive-data](https://eur01.safelinks.protection.outlook.com/?url=https%3A%2F%2Fjournals.plos.org%2Fplosone%2Fs%2Fdata-availability%23loc-human-research-participant-data-and-other-sensitive-data&data=05%7C02%7Cm.jongkees%40erasmusmc.nl%7Ce925bc084456499b648608dd21a5c36b%7C526638ba6af34b0fa532a1a511f4ac80%7C0%7C0%7C638703718795583167%7CUnknown%7CTWFpbGZsb3d8eyJFbXB0eU1hcGkiOnRydWUsIlYiOiIwLjAuMDAwMCIsIlAiOiJXaW4zMiIsIkFOIjoiTWFpbCIsIldUIjoyfQ%3D%3D%7C0%7C%7C%7C&sdata=y2SJj2d%2BYdC519ilsC05VeO9IEFpWo3rbukJotR35bM%3D&reserved=0)) and in the following article: [http://www.bmj.com/content/340/bmj.c181.long](https://eur01.safelinks.protection.outlook.com/?url=http%3A%2F%2Fwww.bmj.com%2Fcontent%2F340%2Fbmj.c181.long&data=05%7C02%7Cm.jongkees%40erasmusmc.nl%7Ce925bc084456499b648608dd21a5c36b%7C526638ba6af34b0fa532a1a511f4ac80%7C0%7C0%7C638703718795605720%7CUnknown%7CTWFpbGZsb3d8eyJFbXB0eU1hcGkiOnRydWUsIlYiOiIwLjAuMDAwMCIsIlAiOiJXaW4zMiIsIkFOIjoiTWFpbCIsIldUIjoyfQ%3D%3D%7C0%7C%7C%7C&sdata=o8peibfuc%2F0acKGhu7f0kgzEg%2FW6n42vs2B7iPJgVMc%3D&reserved=0).

8. Please review your reference list to ensure that it is complete and correct. If you have cited papers that have been retracted, please include the rationale for doing so in the manuscript text, or remove these references and replace them with relevant current references. Any changes to the reference list should be mentioned in the rebuttal letter that accompanies your revised manuscript. If you need to cite a retracted article, indicate the article’s retracted status in the References list and also include a citation and full reference for the retraction notice.

The reference list has been checked and updated.


[Note: HTML markup is below. Please do not edit.]

Reviewers' comments:

Reviewer's Responses to Questions

**Comments to the Author**

1. Is the manuscript technically sound, and do the data support the conclusions?

The manuscript must describe a technically sound piece of scientific research with data that supports the conclusions. Experiments must have been conducted rigorously, with appropriate controls, replication, and sample sizes. The conclusions must be drawn appropriately based on the data presented.

Reviewer #1: Yes

Reviewer #2: Yes

Reviewer #3: Yes

2. Has the statistical analysis been performed appropriately and rigorously?

Reviewer #1: Yes

Reviewer #2: Yes

Reviewer #3: Yes

3. Have the authors made all data underlying the findings in their manuscript fully available?

The [PLOS Data policy](https://eur01.safelinks.protection.outlook.com/?url=http%3A%2F%2Ftrack.editorialmanager.com%2FCL0%2Fhttp%3A%252F%252Fwww.plosone.org%252Fstatic%252Fpolicies.action%2523sharing%2F1%2F010f0193e7ada972-88bd89c2-9e51-4a44-89a5-2e8213c4cf36-000000%2FvJ5ZX07Hhz_1vY4zAygbuh_jyhhQXnODnK4GuI_HTu4%3D190&data=05%7C02%7Cm.jongkees%40erasmusmc.nl%7Ce925bc084456499b648608dd21a5c36b%7C526638ba6af34b0fa532a1a511f4ac80%7C0%7C0%7C638703718795731700%7CUnknown%7CTWFpbGZsb3d8eyJFbXB0eU1hcGkiOnRydWUsIlYiOiIwLjAuMDAwMCIsIlAiOiJXaW4zMiIsIkFOIjoiTWFpbCIsIldUIjoyfQ%3D%3D%7C0%7C%7C%7C&sdata=7dMt4YcgtAnHDCblHAAkUG8lojLoxXeQjYc0b0oeJXc%3D&reserved=0) requires authors to make all data underlying the findings described in their manuscript fully available without restriction, with rare exception (please refer to the Data Availability Statement in the manuscript PDF file). The data should be provided as part of the manuscript or its supporting information, or deposited to a public repository. For example, in addition to summary statistics, the data points behind means, medians and variance measures should be available. If there are restrictions on publicly sharing data—e.g. participant privacy or use of data from a third party—those must be specified.

Reviewer #1: No

Reviewer #2: No

Reviewer #3: Yes

4. Is the manuscript presented in an intelligible fashion and written in standard English?

PLOS ONE does not copyedit accepted manuscripts, so the language in submitted articles must be clear, correct, and unambiguous. Any typographical or grammatical errors should be corrected at revision, so please note any specific errors here.

Reviewer #1: Yes

Reviewer #2: Yes

Reviewer #3: Yes

5. Review Comments to the Author

Please use the space provided to explain your answers to the questions above. You may also include additional comments for the author, including concerns about dual publication, research ethics, or publication ethics. (Please upload your review as an attachment if it exceeds 20,000 characters)

Reviewer #1: This study presents a prospective cohort study in adult people with HIV. The primary outcome was the SARS-CoV-2 spike-specific antibody level at 1, 6, 12, 18, and 24 months after completing a primary COVID-19 vaccination series. The authors compared the antibody kinetics over two years between PWH with a baseline CD4+ T-cell count <200 (n=16) vs. ≥200 (n=432) with a mixed-effects model. The authors conclude that long-term humoral responses were lower in PWH with a CD4+ T-cell count <200 compared to those with a CD4+ T-cell count ≥200.

This is a well-written manuscript presenting important data and emphasizing the importance of individual assessment of the need of COVID-19 vaccination among people living with HIV. See my comments below.

Abstract
Q1. Background: I would consider rephrasing the description of guideline recommendations given that both EACS, BHIVA and CDC are quite united in that priority should be giving those with advanced HIV-infection (low CD4 and detectable viral load). See EACS Guidelines 12.0 page 100, BHIVA ([https://www.bhiva.org/SARS-CoV-2-vaccine-advice-for-adults-living-with-HIV-update](https://eur01.safelinks.protection.outlook.com/?url=https%3A%2F%2Fwww.bhiva.org%2FSARS-CoV-2-vaccine-advice-for-adults-living-with-HIV-update&data=05%7C02%7Cm.jongkees%40erasmusmc.nl%7Ce925bc084456499b648608dd21a5c36b%7C526638ba6af34b0fa532a1a511f4ac80%7C0%7C0%7C638703718795757131%7CUnknown%7CTWFpbGZsb3d8eyJFbXB0eU1hcGkiOnRydWUsIlYiOiIwLjAuMDAwMCIsIlAiOiJXaW4zMiIsIkFOIjoiTWFpbCIsIldUIjoyfQ%3D%3D%7C0%7C%7C%7C&sdata=NWw1obzP4OlZa3YOA81xOCoSzms5LMyLVgGA%2B08hJ%2FQ%3D&reserved=0)) and CDC ([https://www.cdc.gov/vaccines/covid-19/clinical-considerations/interim-considerations-us.html](https://eur01.safelinks.protection.outlook.com/?url=https%3A%2F%2Fwww.cdc.gov%2Fvaccines%2Fcovid-19%2Fclinical-considerations%2Finterim-considerations-us.html&data=05%7C02%7Cm.jongkees%40erasmusmc.nl%7Ce925bc084456499b648608dd21a5c36b%7C526638ba6af34b0fa532a1a511f4ac80%7C0%7C0%7C638703718795777580%7CUnknown%7CTWFpbGZsb3d8eyJFbXB0eU1hcGkiOnRydWUsIlYiOiIwLjAuMDAwMCIsIlAiOiJXaW4zMiIsIkFOIjoiTWFpbCIsIldUIjoyfQ%3D%3D%7C0%7C%7C%7C&sdata=881AgNVg06ZbjwVe%2Fw2rDmz1NvYEYauk1dHh%2F4PNQuE%3D&reserved=0))

Thank you for this suggestion. We have adjusted the background to make clear that guidelines indeed all prioritise PWH with advanced HIV or those who are clinically vulnerable. The background now reads as follows:

*Although guidelines for COVID-19 additional vaccination strategies generally prioritize people with advanced HIV infection, recommendations vary globally, with some countries recommending an annual vaccination for all people with HIV (PWH), while others restrict this to PWH with a CD4+ T-cell count <200 cells per µL.*

Q2. In the method section, lines 30-31, consider including a definition of primary series.

We have added a description of the primary COVID-19 vaccination series used in our participants.

*See line 94-97: Between January and October 2021 participants received the primary vaccination series in accordance with manufacturers’ regulations as part of the Dutch COVID-19 vaccination campaign. The primary vaccination series consisted of two doses of BNT162b2, mRNA-1273, or ChAdOx1-S, or one dose of Ad26.COV2.S.*

Introduction
Q3. Page 5, lines 51-52: Consider rephrasing. Some studies have shown higher mortality, some have not. Suggest adding references that represents both findings. Example: doi: 10.1097/QAD. 0000000000003129; doi: 10.1111/hiv.13174; doi: 10.1111/hiv.13515.

Thank you for your suggestion. We referred to a systematic review that we believed represents the highest level of evidence on this topic. This review included 27 studies on COVID-19 mortality in people with HIV (PWH), and one of the suggested references is also included in the review. The review found a slightly higher mortality risk in PWH compared to immunocompetent individuals (RR 1.20, 95% CI 1.05-1.36), and we have included the relative risk (RR) and confidence interval in the introduction of our revised manuscript.

Regarding the suggested references:

- **doi: 10.1097/QAD.0000000000003129**: This study is included in the systematic review.
- **doi: 10.1111/hiv.13174**: This reference is not included in the systematic review, but earlier results from the same author, based on a smaller cohort, are included in the review.
- **doi: 10.1111/hiv.13515**: This study is not included in the systematic review.

Q4. Page 5, line 63, first sentence, suggest adding references regarding earlier studies with follow-up.

We have added three references of earlier COVID-19 vaccination studies in PWH with follow-up.

Q5. Suggest adding a sentence in the Introduction that states current guidelines on vaccination in people with HIV (see examples above). In particular guidelines from the country the study was conducted in.

We have added the following sentence to the last paragraph of the introduction to address current guidelines on COVID-19 vaccination for people with HIV (PWH):

The lack of long-term data is reflected in the inconsistency of national guidelines regarding COVID-19 booster vaccination strategies for PWH. While the Centers for Disease Control and Prevention in the US recommend a yearly booster vaccination for PWH with a CD4+ T-cell count <200 cells per µL, European guidelines generally recommend yearly vaccination for all PWH, irrespective of their CD4+ T-cell count.

Method
Q6. Primary outcome is stated as: The primary outcome was the level of S1-specific antibodies in PWH at 1, 6, 12, 18, and 24 months after primary vaccination.
Perhaps it should be clarified somehow “primary vaccination including/adjusted for the following boosters”? Because you do in fact account for the boosters?

For the primary outcome, we did not adjust for booster vaccinations. However, as a secondary endpoint, we accounted for different variables in the model, including time since the last COVID-19 vaccination dose or last SARS-CoV-2 infection.

Q7. In the subheading Clinical procedures, it says that data on comorbidities and co-medications were collected. I suggest presenting this in Table 1 and consider taken this into consideration in the statistical analysis or state why this was not deemed necessary.

Thank you for addressing this as this was not fully correct. Comorbidities were not systematically collected, so we have removed this from the subheading "Clinical procedures." It was only collected if participants had a diagnosis of solid organ neoplasia or haematological malignancy within 12 months prior to the primary vaccination series. This is added to Table 1 and adjusted in the methods section. Regarding co-medication, only the use of immunosuppressive therapy was recorded. In the methods section co-medication is replaced by use of immunosuppressive therapy and this information has also been included in Table 1.

Q8. Suggest stating more clearly the definition of Primary series vaccination in the Clinical procedures section where you state the different types of possible primary vaccinations. “Participants received two doses of BNT162b2, mRNA-1273, or ChAdOx1-S, or one dose of Ad26.COV2.S between January and August 2021 according to manufacturer’s regulations as part of the Dutch COVID-19 vaccination campaign”

Thank you, we have adjusted this sentence for clarity:

Between January and October 2021 participants received the primary vaccination series in accordance with manufacturers’ regulations as part of the Dutch COVID-19 vaccination campaign. The primary vaccination series consisted of two doses of BNT162b2, mRNA-1273, or ChAdOx1-S, or one dose of Ad26.COV2.S.

Q9. In the Statistical analysis you state among variables added to the model “time since the last COVID-19 vaccination dose or last SARS-CoV-2 infection”. I guess this refers to the boosters? Perhaps it would be good to clarify that boosters were considered in the model.

You are correct that "time since the last COVID-19 vaccination dose or last SARS-CoV-2 infection" primarily refers to booster doses. A few participants did not receive a booster, so for them, it refers to the time since the primary vaccination regimen. For the majority of participants, however, it refers to the time since the last booster dose (or infection). To clarify, we have updated the text to read: "and time since the last COVID-19 (booster) vaccination dose or last SARS-CoV-2 infection."

Table 1:
Q10. History of a third SARSCoV-2 infection is marked with a b but it should perhaps be c?

You are correct; it should have been marked with both "b" and "c." We have made the necessary correction and added the superscript "c."

Q11. In the first column, including all PWH, n=448. The sum in the column of “Covid-19 vaccines received” is just 403. Do you have missing data? Suggest updating table 1 so the numbers add up.

Yes, you are correct that there is missing data for the COVID-19 vaccines received and for the history of SARS-CoV-2 infection. We have indicated this with the letter "b" and excluded the missing data from the denominators, as noted in the table's footnote. The percentage of participants of whom we did not know the number of received vaccinations is not equally distributed between the two groups (no unknown number of vaccines received in the <200 groups vs 45 unknown number of vaccines received in the >200 group). If we would include a row with ‘unknown number of vaccinations’, the other percentages in the >200 group will look comparatively lower. For comparative reasons, we have therefore decided to exclude the missing data from the denominators.

Q12. The same goes for History of at least one SARS-CoV-2 infection. The numbers do not add up. Missing data?

Yes, you are correct. There is missing data for the history of at least one SARS-CoV-2 infection, as mentioned in our response to Q11.

Q13. Same for: Dominant variant in the Netherlands at the time of first reported SARS-CoV-2 infection. Missing data?

Yes, the same issue applies to the dominant variant in the Netherlands at the time of the first reported SARS-CoV-2 infection. Missing data has been accounted for as described in our response to Q11.

Results
Q14. Page 15, lines 215-216: “Of these, one participant– a 61-year-old male – was admitted to the intensive care unit. His most recent CD4+ T-cell count was 316 cells per µL, and his nadir CD4+ T-cell count was 50 cells per µL.” This maybe reveals too much information about one patient and could potentially violate his integrity? This needs to be considered through the whole paragraph.

Thank you for addressing this. We agree that it is important to maintain patients’ confidentiality. We now left out the nadir CD4+ T-cell counts and categorized the most recent CD4+ T-cell counts in the following groups: <350, <500 and ≥500.

Discussion
Q15. The Discussion is quite short and could benefit from a more elaborate discussion of its findings and how it compares to other studies. For example, you state that this is the only study “this is the first longitudinal study in PWH extending beyond one year of follow-up after COVID-19 vaccination”. What is the follow-up time of the most recent studies?

Thank you for your suggestion. We have now included three references to studies on COVID-19 vaccination in people with HIV (PWH) with the longest follow-up in both the introduction and the discussion. The longest follow-up in the most recent studies is 12 months after primary vaccination in two of these studies, and the third study has a follow-up of 11 months after booster vaccination. As mentioned in the discussion, these previous studies have shown comparable humoral responses between PWH and HIV-negative controls. However, it should be noted that the number of PWH with a low CD4+ T-cell count was very limited: 7 and 4 PWH with a CD4+ T-cell count <200 in two studies, and 11 PWH with a CD4+ T-cell count <300 in the third study.

Q16. Page 16, 222-225; here you conclude lower antibody levels after COVID-19 vaccination, as mentioned in Q9 it could be clearer what you mean by “COVID-19 vaccination”. Primary or primary including/adjusted for boosters?

Thank you. We are referring to the two years following a primary COVID-19 vaccination series. This analysis was not adjusted for booster doses. To clarify, we have updated the text by changing “after COVID-19 vaccination” to “after a primary COVID-19 vaccination series.”

Q17. You state in line 224 that the booster rates were comparable, but they are quite different in Table 1 between groups (primary + 2 boosters: 26, 7% vs 35,1%)?

Thank you for your comment. We acknowledge that the booster rates are not exactly the same between the groups (26.7% vs. 35.1% for the primary series + 2 boosters, respectively). However, it is important to note that the group with a CD4+ T-cell count <200 cells per µL did not receive fewer booster doses compared to the group with a CD4+ T-cell count >200 cells per µL, e.g. a little higher percentage had primary vaccination + 3 boosters. Specifically, the median number of booster vaccines was similar between both groups (2, IQR 1–3). This suggests that the difference in antibody levels is not due to the number of booster doses received, as the distribution of boosters is relatively similar across both groups.

Q18.Page 17, lines 249-251, please include references to these section

The references have been included to the section.

Limitations:
Q19. Suggest adding to the conclusion that the majority of your sample are men and how that effects generalizability.

We have added the following sentence to the limitations section:

*"The majority of participants in our study were male, although the sex distribution was representative of the Dutch population of PWH and comparable between the two groups."*

Reviewer #2: Dear Dr. Roukens,

I appreciate the opportunity to review this interesting and important article longitudinal assessment of COVID-19 vaccine immunogenicity in people with HIV stratified by CD4+ T-cell count in the Netherlands: a two-year follow-up study. I enjoyed reading the manuscript. I commend the work on several strengths including:

1. Addressing the immunogenicity in PWH with CD4 < 200 and showing that this group needs to be prioritized. This is especially important during an epidemic and in low resource setting where prioritization is important.

2. Large sample size drawn from multiple centers, along with comprehensive follow-up

3. Longitudinal and prospective data collection, for an impressive duration of more than a year.

Considering the strengths of the manuscript, I noted a few areas where additional clarity would enhance its overall impact. Specifically, the paper could be further strengthened by incorporating more detailed information on the following points:


1. If the author could clarify the power calculations to clarify the need to include more participants, and the rationale for the inclusion of participants with a CD4 cell count of <500, along with precautions taken to minimize any potential bias with retrospective inclusion of participants. Furthermore, the inclusion of retrospective participants was not reflected in the figure. We would appreciate more clarification on the inclusion of the participants in the attached figure as well.

Those additional participants with a CD4 count <500 were selected via treating HIV physicians in one of the participating centres (Erasmus MC). All patients of the Erasmus MC with a CD4 count <500 were asked if they want to participate in this study. The rationale for the inclusion of participants with a CD4 cell count <500 was based on the existing literature at that time. As this manuscript focuses on the impact of CD4 count on antibody levels over time, it was essential for us to not only improve the length of follow-up compared to previous studies but also the number of included participants with a low CD4 count compared to the existing literature. To address the potential for bias from including retrospective participants, precautions were taken to minimize any potential bias. Data regarding previous COVID-19 infections and/or vaccination were collected after participants provided written informed consent, and potential previous antibody measurements were unknown at the time of inclusion. Furthermore, no analyses had been performed at the time the additional participants were included. In the supplementary materials, we have included S1 Figure (also referred to in the text), in which all participants that were included retrospectively are highlighted as darker triangles. We have included this description in the clinical procedures section of the Methods.

2. In the baseline results, there is a mention of a questionnaire, it is the first mention of a it being used in the study and was not mentioned in the methods. Please include it in the methods section as well with details as to how it was administered.

The last sentence of the methods section clinical procedures states:
*Dates and types of previous COVID-19 vaccinations, as well as dates of intercurrent SARS-CoV-2 infections, including the test type, whether PCR or rapid antigen test, and infection severity, were obtained through yearly online questionnaires and telephone surveys.*

3. In the limitations, if you could also include the retrospective inclusion of participants could influence the results and precautions that were taken.

We have added the retrospective inclusion of participants as limitation in the discussion:

*To increase the number of participants with a low CD4+ T-cell count, 16 participants with a CD4+ T-cell count < 500 were retrospectively included prior to the first analysis. Although all eligible PWH in one of the HIV treatment centres were invited to participate and antibody levels, previous COVID-19 vaccinations, and SARS-CoV-2 infection history were unknown at the time of inclusion, retrospective inclusion could introduce selection bias.*

4. Additionally, if the authors could comment in manuscript, in the group with pre vaccination CD4 of >200, the participants who had a nadir of CD4 < 200 vs. the participants who had a nadir of CD4>200, would benefit from a booster. If it is not possible to calculate, can it be added to the limitations or the discussion (as per author’s discretion).

We have included a new figure (S2 Fig.) showing antibody concentrations in PWH stratified by nadir CD4 groups (<200 vs ≥200). There is no difference in antibody concentrations over time between these two groups. Also, in the second model which investigates variables associated with the antibody levels over time, no association was found between nadir CD4 count and the S1-specific antibodies over time.

5. If the authors could clarify what was the cutoff age used to classify “higher age”, as referred to in the manuscript.

We did not use a specific cutoff age to classify "higher age" in our analyses. Instead, age was treated as a continuous numerical variable, allowing us to examine its effect across the entire age range of participants. This approach provides a more nuanced understanding of how age influences the outcomes, rather than categorizing participants into arbitrary age groups. We have added this clarification to the methods section.

Best,

Soahum

Reviewer #3: Overall, I find this article interesting and valuable. The proposed study addresses an important and novel research question. The writing is easy to understand, and the flow of the text is good; the topic is clinically relevant and has ethics committee approval; there are some spelling and grammar errors that need revision.
